# Understanding Footwear Needs: A Conceptual Review

**DOI:** 10.1002/jfa2.70089

**Published:** 2025-10-11

**Authors:** Thanaporn Tunprasert, Stewart C. Morrison, Leonard Henry Joseph, Paula Kersten

**Affiliations:** ^1^ School of Education, Sport and Health Sciences University of Brighton Brighton UK; ^2^ School of Life Course and Population Sciences King's College London London UK; ^3^ School of Applied Health Sciences University of Suffolk Ipswich UK

**Keywords:** biopsychosocial outcomes, shoe choice, shoe comfort, shoe design, shoe interventions

## Abstract

**Introduction:**

This conceptual review is the first work to explore the concept of footwear needs. The review draws upon multidisciplinary literature to synthesise and formulate the first iteration of conceptual understanding of footwear needs.

**Methods:**

A systematic search was performed and through a screening process, 81 studies were included in this conceptual review. The included studies were analysed using the pragmatic utility meta‐synthesis method to develop conceptual understanding and framework. The review process was further strengthened through expert consultation.

**Results:**

The definition of footwear needs is defined as ‘footwear requirements for the well‐being of a person’. Preconditions of footwear needs include characteristics related to the person, product (footwear) and circumstances. Attributes of footwear needs encompass physical (*fit for foot* and *fit for purpose*), safety (*footwear safety* and *financial safety*), social (*fit for person* and *fit for society*) and emotional (*increase positive emotions* and *decrease negative emotions*) needs. Outcomes of footwear needs are related to physical, psychological and social aspects of a person. The conceptual framework of footwear needs illustrates the relationships between various factors underlying footwear needs. Footwear comfort is also identified as an allied concept and a potential outcome when footwear needs are achieved.

**Conclusion:**

This conceptual review provides valuable insights into footwear needs, offering a foundation for future research and practical applications in footwear assessment, education and interventions.

## 1 Introduction

Footwear can cause or alleviate foot problems, depending on whether it meets an individual’s needs [1, 2, 3, 4]. The functional, expressive and aesthetic (FEA) consumer needs model identifies functional, expressive and aesthetic as three main needs for apparel [5]. Although the FEA model can be used to aid apparel design, it does not explore why individuals consider these factors. Specific to footwear, there are some qualitative studies exploring footwear needs in workers in standing environments [1] and older adults [4] found that fit, comfort, ease of use, price and personal preferences might be the key influencing factors. Despite the wealth of knowledge, substantial portion of the population (63%–72%) wear unsuitable footwear daily [6]. There is also evidence to suggest that individuals often wear unsuitable footwear despite being aware of more appropriate options [7]. This highlights the importance of exploring footwear needs beyond their physical characteristics.

Footwear comfort is a major determinant in footwear selection [8, 9]. Comfort can be assessed based on sensation and perceptions from wearing footwear [10]. It integrates physical and psychological aspects of footwear selection [11]. However, comfort perception is not always a reliable nor valid indicator for achieving footwear needs. For example, people with diabetes with sensory neuropathy may be unable to sufficiently determine footwear comfort level linked to its physical performance [12]. In addition to physical factors and comfort, psychological and social aspects also influence perceptions of footwear suitability. Social situations or work requirements can override personal preferences [13]. Cost has also been cited as another key determinant [14].

Research on footwear needs is fragmented, often focusing on specific factors in isolation and lacking consideration of the interplay between physical, psychological and social factors. Studies are typically conducted on specific demographic groups [15, 16, 17], limiting their generalisability. There is also ambiguity in defining footwear needs. A conceptual review can integrate these insights, offering a broader perspective on the motivations behind footwear choices across different populations. This review aims to develop a conceptual framework encompassing physical, psychological and social aspects of footwear needs and establish a clear definition to guide future research.

## 2 Methods

### 2.1 Overall Approach

The conceptual review followed the pragmatic utility approach proposed by Morse and colleagues [18, 19, 20]. This meta‐synthesis method analyses existing literature to advance a concept using available literature across several disciplines [21]. The pragmatic utility approach uses the anatomy of concept to analyse conceptual data, which includes definition, boundaries, attributes, preconditions and outcomes [20, 22].

### 2.2 Systematic Search and Screening Process

A systematic search was performed via PubMed, Scopus and Web of Science (WoS) using the SPIDER search strategy [23]. An initial scoping search revealed limited publications using the terms ‘footwear needs’ and its direct variations. ‘Footwear needs’ were classed a partially mature concept, where understanding is fragmented and dispersed across multidisciplinary literature involving overlapping constructs [20, 22]. Thus, the scoping search result was unsurprising. To enhance the sensitivity of the search and ensure the inclusion of relevant studies, the search terms were therefore broadened to encompass keywords related to footwear needs, choice and design, with the following rationale:

- Footwear choice to represent footwear needs from wearers' perspectives
- Footwear design to represent footwear needs from clinicians', researchers' and footwear designers' perspectives

The complete information on the SPIDER search strategy and search terms can be found in Supporting Information S1. Following the searches, results were imported into Microsoft Excel for removal of duplications, screening of titles and abstracts using the inclusion criteria. Full articles were obtained and imported to Zotero (v. 6.0.36) for further eligibility screening. Articles were included if at least one of their research objectives was to:

1. Provide an understanding of the concept(s) relevant to footwear needs, choice or design.
2. Review concepts relevant to footwear needs, choices or design.
3. Develop or apply a questionnaire for footwear needs, choices or design.

Only the articles that met the inclusion criteria after the eligibility screening were used in the conceptual analysis. Studies were excluded if they were not in English, not related to footwear, not performed in human participants and/or were performed with children.

### 2.3 Conceptual Analysis

The full text for all articles meeting the inclusion criteria was reviewed by T.T. and discussed with S.C.M., L.H.J. and P.K. The conceptual analysis followed the established pragmatic utility approach [20]. The explicit and implicit statements relevant to footwear needs within the included articles were coded in NVivo software (version 1.7.1) and sorted accordingly by the anatomy of concept (i.e., definition, boundaries, attributes, preconditions and outcomes). Subsequently, the collated codes were synthesised through analytic questioning into pertinent themes. Based on the pragmatic utility approach, the analytical questions were a series of questions addressing clarity, significance, depth, breadth, data‐driven/data‐transformative qualities, comprehensiveness, comparison and balance. These guided the coding and categorisation process, helping to minimise arbitrary decisions and enhance consistency. The themes were analysed for relationships to create a conceptual framework allowing for an improved presentation of the concept. The conceptual analysis was strengthened by regular team discussions and the integration of diverse disciplinary perspectives of the authors to maintain reflexivity.

### 2.4 Face Validity

The face validity of the conceptual review was determined through expert consultation, which is a common method for conceptual reviews [24, 25, 26]. Experts were individuals with extensive research and/or practical experience in footwear. Five experts were identified through the Footwear Biomechanics Group and Special Advisory Groups of the Royal College of Podiatry. The conceptual framework and its components were presented to the experts for critical review through a one‐to‐one meeting between the experts and lead author (T.T.). Experts were asked to review the framework and determine whether it appeared appropriate and relevant to the concept it aimed to represent. Their feedback was used to refine elements, such as terminology, and structure, and to enhance overall coherence and usability.

## 3 Results

### 3.1 Eligible Studies and Overall Characteristics

**FIGURE 1:**
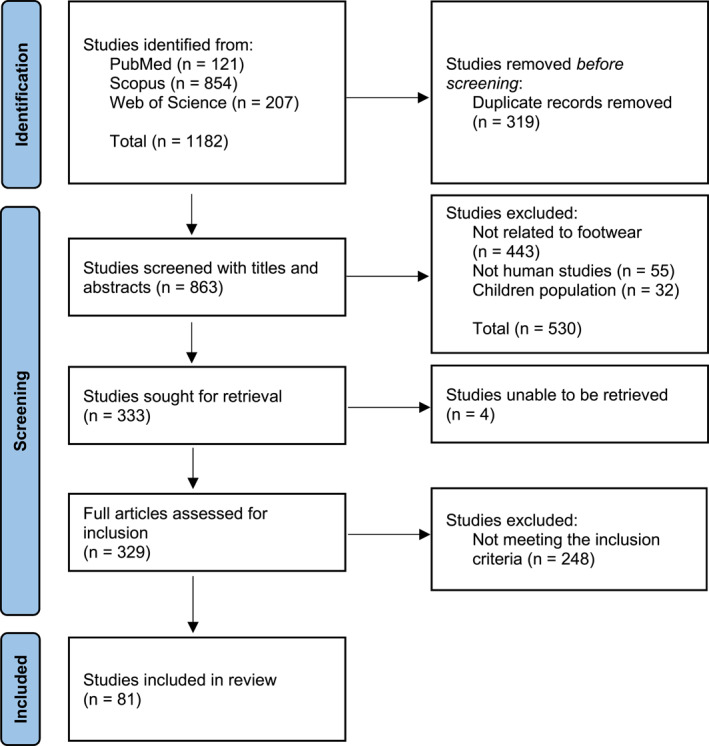
PRISMA flow diagram [27].

Eleven Thousand Eight Hundred Twenty articles were initially identified from PubMed (*n* = 121), Scopus (*n* = 854) and WoS (*n* = 207; Figure 1). Following the screening processes, the total number of articles included for analysis in the conceptual review was 81. The complete list of included articles and their mapping to the anatomy of the concept can be found in Supporting Information S2. Only the most relevant articles are cited in this conceptual review as references.

### 3.2 Anatomy of the Concept of Footwear Needs

The themes relevant to the footwear needs concept are collated under the anatomy of concept sections (i.e., definition, boundaries, attributes, preconditions and outcomes).

#### 3.2.1 Definitions

None of the articles included an explicit definition of footwear needs. However, based on the implicit discussion of footwear needs [1, 4, 16, 28], we derived a definition of footwear needs as ‘footwear requirements for the well‐being of a person’.

#### 3.2.2 Boundaries

Boundaries are characteristics that allow the concept to be differentiated from other allied concepts. The main allied concept identified in this conceptual review of footwear needs is footwear comfort [9, 11, 29, 30, 31, 32] and the characteristics of this are collated in Table 1, alongside the relevant characteristics of footwear needs for comparison.

**TABLE 1:** Boundaries between footwear needs and footwear comfort.

Footwear needs	Footwear comfort
Footwear needs are about a person's requirements related to footwear. Can be determined based on information about a person. As needs are about a person, the information can be applied to multiple footwear.	Footwear comfort is about a person's perception of each footwear. Can only be determined after an interaction (e.g., physically and visually) between a person and each piece of footwear. As comfort is about each piece of footwear, the information may not be transferable to other footwear.

#### 3.2.3 Preconditions

**FIGURE 2:**
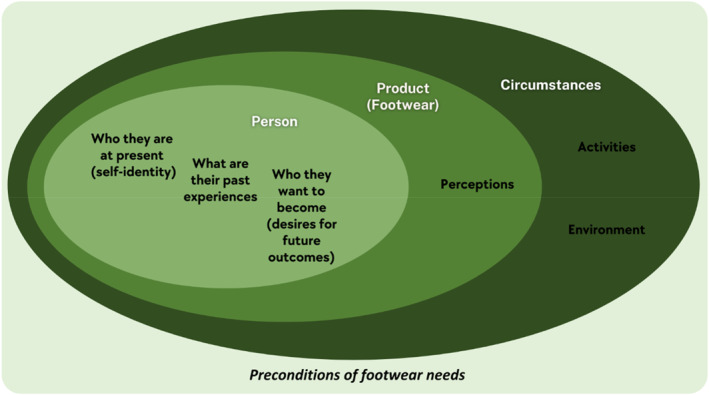
An overview of preconditions of footwear needs.

Themes relevant to preconditions of footwear needs (i.e., characteristics required for ‘footwear requirements for the well‐being of a person’ to occur) are found were reported in 59 out of the 81 included studies (73%). Three themes were established: person, product (footwear) and circumstances. The relationships between the three themes are illustrated in Figure 2.

##### 3.2.3.1 Person

To understand a person’s footwear needs, we must first understand the person, focusing on their self‐identity, past experiences and future intentions.

A person's self‐identity related to their footwear needs are influenced by factors such as age [15, 33, 34], country of origin [35, 36, 37], gender [38, 39, 40], foot shape [13, 41, 42], health conditions [2, 17, 43] and socioeconomic status [30, 38, 44]. The degree to which each characteristic influences their self‐identity varies. For example, foot shape due to congenital deformity or health conditions can significantly impact self‐identity [45]. Both physical [40, 43, 46, 47] and emotional [37, 48, 49] past experiences with footwear influence perspectives on footwear needs. For instance, a fall may lead someone to avoid certain footwear, believing that it caused the fall [46]. Older adults tend to stick to familiar footwear, making past experiences a stronger influence [39]. Emotional experiences, such as embarrassment from wearing ‘ugly’ footwear, especially in those with health conditions requiring specialised footwear, also play a role [34, 37, 48, 49]. Desires for future outcomes also influence footwear needs. Older adults often seek to maintain independence [42, 43, 50], whereas athletes aim for improved performance and injury prevention [51, 52]. Additionally, individuals may want to express themselves through their footwear, reflecting how they wish to be perceived by others [34, 53, 54].

##### 3.2.3.2 Product (Footwear)

The next layer of preconditions for footwear needs is the product itself, particularly individuals’ perceptions of footwear, which encompass general attitudes, beliefs and impressions. These perceptions fall into three main categories: value, functionality and suitability [1, 14, 17, 33, 40, 46]. They are influenced by the inner layer of preconditions (i.e., person), visual perceptions [33, 47, 55, 56] and brand identity [34, 39, 57]. Visual perceptions (e.g., style, design, materials and colours) lead to assumptions about comfort, quality, weight, functions, performance and identity representations [55, 56]. Brand identity perceptions align with how brands position themselves in the market [34, 57]. These perceptions can strongly influence footwear choices, often outweighing the actual qualities or features of the shoes.

##### 3.2.3.3 Circumstances

The outermost layer of preconditions involves a person's circumstances, including their activities [1, 56, 58, 59, 60, 61] and environment [34, 36, 37, 41, 62]. Profession, role(s) and hobbies dictate the activities that individuals engage in, often requiring specific footwear, sometimes mandated by laws (e.g., safety footwear) [1, 61]. Environment refers to external conditions such as climate and weather [38, 41, 58].

#### 3.2.4 Attributes

**FIGURE 3:**
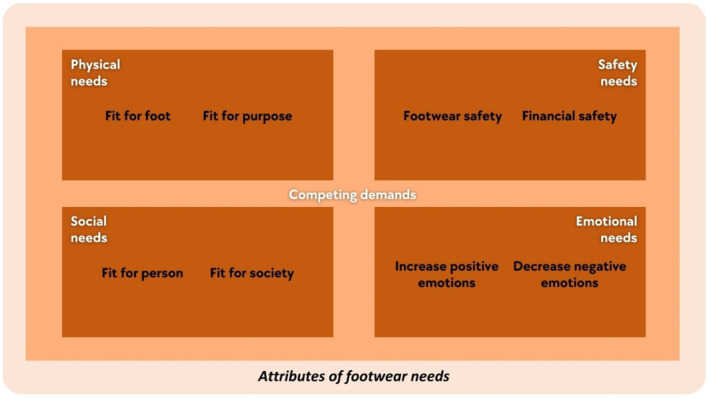
An overview of attributes of footwear needs.

Themes relevant to attributes of footwear needs (i.e., characteristics required for ‘footwear requirements for the well‐being of a person’ to occur) are in 73 out of the total 81 included studies (90%). Four themes were identified: physical needs, safety needs, social needs and emotional needs (see Figure 3). Each theme also has two relevant sub‐attributes. All themes have competing demands because of a person's preconditions of footwear needs.

##### 3.2.4.1 Physical Needs

The physical aspects of footwear needs have two sub‐attributes: fit for foot and fit for purpose. At the most fundamental level, the footwear must be suitable for the wearer's feet (i.e., *fit for foot*). The footwear must also fit the intention of use (i.e., *fit for purpose*).

The determinants of *fit for foot* include width [63, 64, 65, 66], depth [33, 38, 67, 68, 69], length [32, 48, 70, 71] and circumference [15, 32, 48, 72, 73, 74]. Width and depth typically refer to the toe box, whereas length measures the longest part of the shoe [63, 64, 69, 75, 76]. Circumferences can be measured in various parts of footwear (e.g., at the forefoot, midfoot and rearfoot) [72, 73]. A match between foot and shoe measurements typically indicates a good fit [6, 13, 73]. These determinants are generally consistent over time, except when foot shape changes due to health conditions. The determinants of *fit for purpose* include weight [16, 41, 51, 52, 60, 74], climate control [35, 77, 78, 79, 80], cushioning [61, 65, 81, 82, 83], ease of use [39, 42, 49, 58], flexibility [44, 46, 57, 74] and appropriate materials [11, 31, 69]. The purpose of footwear can vary based on activities, so footwear needs may involve multiple sets of determinants, each suited to different activities.

##### 3.2.4.2 Safety Needs

The second attribute of footwear needs is safety needs (i.e., *footwear safety* and *financial safety*). *Footwear safety* includes grip/slip resistance [58, 77, 84], fastenings [11, 46, 47], protective features [1, 58, 74] and shoe stability [15, 28, 75]. Safety needs depend on a person’s activities, environment and their belief in the required level of protection. For *financial safety*, value for money [1, 14, 30, 85] and affordability [36, 39, 41, 71] are the key determinants. Value for money is the judgement of whether the benefits of the footwear justify the cost, whereas affordability is whether the price fits within a person’s financial capacity.

##### 3.2.4.3 Social Needs

The third attribute of footwear needs is social needs, with two sub‐attributes of *fit for a person* and *fit for society*. For footwear to be fit for the person, it needs to be aligned with the person's self‐image and self‐expression. Footwear must align with self‐image and self‐expression. Characteristics include colour [39, 48, 62], style (distinctive aesthetic approach) [14, 34, 50, 86] and design (specific construction) [54, 70, 87]. Fit for society is a desire for social acceptance [37, 70, 86]. The desire could be related to the overall society or different social groups that the person belongs to or wishes to belong to.

##### 3.2.4.4 Emotional Needs

The fourth attribute of footwear needs is emotional needs. There are two sub‐attributes which are *increased positive emotions* (e.g., joy, happiness and fulfilment) and *decreased negative emotions* (e.g., shame, embarrassment and self‐consciousness) [14, 48, 49, 53]. Footwear must become a medium to increase positive emotions and decrease negative emotions simultaneously.

#### 3.2.5 Outcomes

**FIGURE 4:**
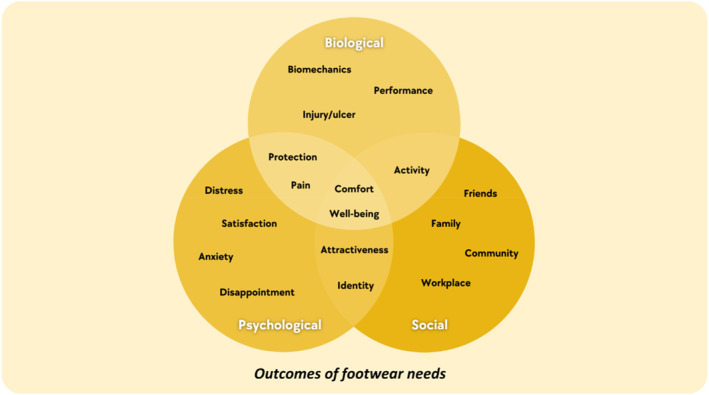
An overview of outcomes of footwear needs.

Outcomes relevant to footwear needs (i.e., outcomes following an application of ‘footwear requirements for the well‐being of a person’) were identified in 65 out of the 81 included studies (80%) and are mapped to the biopsychosocial model [88] (Figure 4). The list of outcomes in the framework (Figure 4) is not exhaustive but demonstrates the most common outcomes from the included articles. See Supporting Information S2 for the complete list of references.

#### 3.2.6 Relationships Between Preconditions, Attributes and Outcomes

**FIGURE 5:**
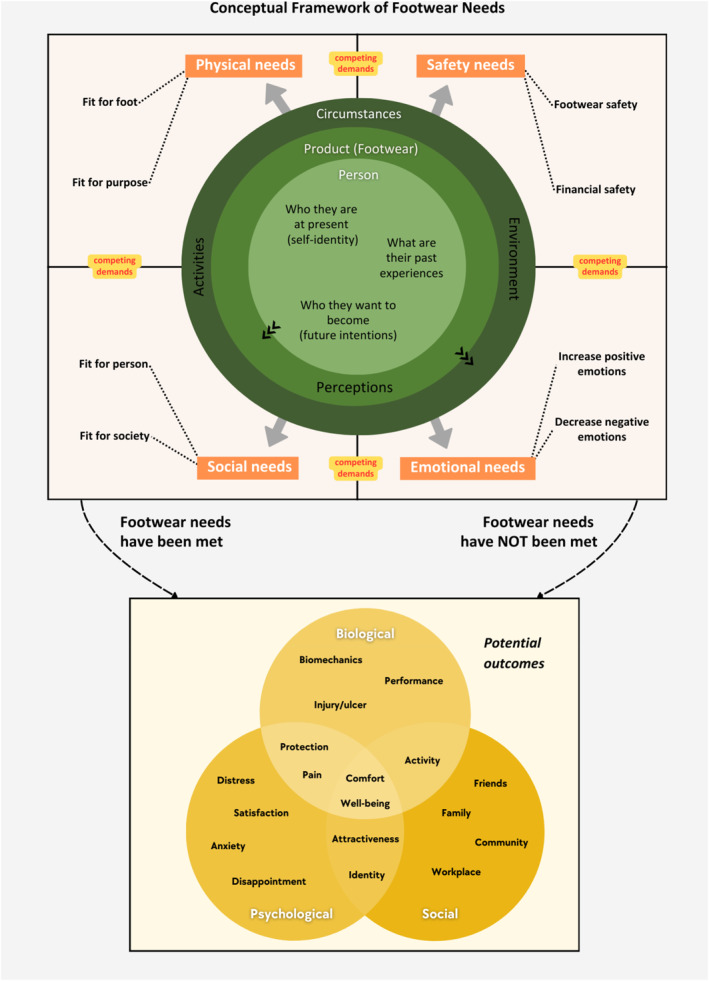
A conceptual framework of footwear needs.

The conceptual framework of footwear needs (Figure 5) demonstrates the relationships between the domains: preconditions, attributes and outcomes. At a conceptual level, the preconditions determine the attributes of footwear needs and whether the footwear needs have been met results in the relevant outcomes.

For example, an individual who felt like they had to wear therapeutic footwear for their foot condition despite feeling self‐conscious about the style of footwear (preconditions) may later develop a heightened emotional need for footwear that helps reduce negative emotions such as discomfort, shame or anxiety (attributes). This emotional need becomes central to their future footwear choices, where feeling confident, comfortable and socially accepted becomes as important as physical fit. When they eventually find a pair of shoes that align with these emotional needs (i.e., their footwear needs are met), they experience emotional satisfaction and improved well‐being (outcome).

## 4 Discussion

This conceptual review is the first work to explore the concept of footwear needs. Using the pragmatic tility framework [20], the conceptual understanding has been analysed according to the concept’s anatomy: definition, boundaries, attributes, preconditions and outcomes. The footwear needs conceptual framework has been developed to provide an overview of the relationships between different aspects of the concept (Figure 5).

Footwear needs are crucial in the treatment and prevention of many lower limb conditions [72, 89]. Traditionally, ‘footwear needs’ often referred to individuals' considerations in the footwear selection process [1, 4]. However, this review redefines ‘footwear needs’ as essential requirements for achieving well‐being, encompassing physical, emotional, social and financial aspects. Unmet footwear needs can compromise overall well‐being (Figure 5). This conceptual definition is a starting point for further theoretical development and empirical validation of the concept in future research.

Clinicians must recognise that patients’ perspectives of their footwear needs are shaped by their preconditions (Figure 2). These preconditions include factors related to the person, perceptions of footwear and circumstances. For instance, an individual still negotiating their self‐identity may struggle to articulate their footwear needs, such as whether they accept or hide their feet [40]. Understanding these perspectives can guide appropriate footwear recommendations. Another example is related to desires for future outcomes. Not all individuals have clear future intentions, which can obscure their clarity of footwear needs. Clinicians can use this as an opportunity to set goals with patients, potentially altering their perceptions of footwear and reducing resistance to change. If an individual has strong self‐beliefs, changing their perceptions of footwear can be challenging. In such cases, addressing the outermost layer of preconditions (i.e., circumstances) may be more effective as it is less tied to the person’s core identity. Once footwear needs based on circumstances are negotiated, inner layers can be addressed. For example, discussing footwear requirements related to an individual’s job (i.e., circumstances) can lead to considering other footwear needs based on personal perceptions and values. If satisfactory outcomes are achieved, no further action is needed. However, if issues, such as pain persist, clinicians can discuss alternative footwear options that may initially conflict with the patient’s perceptions. This iterative process can be repeated with the inner layers of preconditions until a satisfactory outcome of footwear needs is achieved.

Clinicians should avoid prioritising their perspectives to prevent conflicts. Although clinicians often focus on physical aspects (e.g., size and shape), patients may prioritise aesthetics or price [90]. It also becomes apparent, particularly from the qualitative studies included in this conceptual review [34, 37, 50, 86] that not all individuals are consciously aware of their footwear needs. Initially, patients may be able to describe preferences but need time to reflect on underlying reasons. This conflict and the time required for individuals to articulate their needs can create tensions. Using the attributes of footwear needs (Figure 3) as a guide for footwear discussion can help align clinicians’ and patients’ perspectives and prioritise competing demands. Clinicians may also be able to use the examples of outcomes (Figure 4) as part of goal setting process of their management plan.

Footwear comfort is frequently cited as important in footwear selection [17, 47, 83, 91]. However, this review proposes that ‘comfort’ is an outcome rather than an attribute of footwear needs. When footwear needs (i.e., ‘footwear requirements for well‐being’) are met, individuals experience comfort. As per comparison in Table 1, footwear needs are about the person's requirements related to footwear whereas footwear comfort is about the person's perception about each footwear. Although studies on footwear comfort often emphasise physical aspects [11], comfort also encompasses mental relief and contentment [92]. Thus, comfort is an important outcome for evaluating whether footwear needs are met, but footwear assessment and education should focus on the underlying factors (i.e., preconditions and attributes of footwear needs) to improve the overall well‐being. For instance, an older adult may find slippers ‘*very comfortable*’ due to their soft materials and ease of use, which promote independence. Clinically, these slippers may lack support and pose safety risks. Instead of opposing the comfort outcome, clinicians should explore the reasons behind it (e.g., soft materials and independence) and suggest alternatives that provide similar comfort while offering better support and slip resistance. We recognise that there is not yet a universally accepted definition of footwear comfort. Thus, this conceptual understanding of boundaries between footwear needs and footwear comfort may continue to develop as more research emerges.

This review has synthesised existing knowledge on footwear needs but has limitations. Most studies involved specific populations (e.g., patients with arthritis, athletes), potentially biasing results. Non‐English studies were excluded which led to only a small proportion of included articles (8 articles, approx. 10%) being conducted in non‐Western countries (e.g., China, India, Japan, Singapore and Turkey). This limits the cultural generalisability of the findings. More research in diverse and general populations, and non‐Western countries is needed to further enhance the framework’s validity and applicability. In practice, the complexity of footwear needs may hinder clinicians’ use of the framework due to time constraints. Developing a simplified assessment tool based on this framework could bridge the gap between theory and practice, making the concept more accessible and easier to apply. Lastly, it is important to note that conceptual development is considered as an evolutionary process [93]. Therefore, this conceptual review should not be regarded as a definitive conclusion, but as a foundation for ongoing conceptual exploration in this area. Examples of more recent studies related to footwear needs which could be considered as part of future conceptual review development include [94, 95, 96, 97].

## 5 Conclusion

This conceptual review lays the groundwork for understanding footwear needs, covering definition, boundaries, preconditions, attributes and outcomes. The conceptual framework illustrates the relationships between these components, aiding in footwear assessment, interventions, recommendations and education to enhance well‐being. Further research is needed to improve the framework’s cultural validity and applicability to diverse populations. Additionally, developing practical tools based on this framework could enhance its accessibility and usability in real‐world scenarios.

## Author Contributions

**Thanaporn Tunprasert:** conceptualization, formal analysis, investigation, methodology, project administration, visualization, writing – original draft, writing – review and editing. **Stewart C. Morrison:** methodology, supervision, validation, writing – review and editing. **Leonard Henry Joseph:** methodology, supervision, validation, writing – review and editing. **Paula Kersten:** methodology, supervision, validation, writing – review and editing.

## Conflicts of Interest

The authors declare no conflicts of interest.

## Supporting information


Supporting Information S1

Supporting Information S2

## Data Availability

No primary data were used. The supplementary files can find information relevant to the systematic search and conceptual analysis.
