# Supplementary material for: Understanding Footwear Needs: A Conceptual Review
Source: J Foot Ankle Res. 2025 Oct 11;18(4):e70089. doi: 10.1002/jfa2.70089 (PMC12515056; doi:10.1002/jfa2.70089)
Supplement: Supplementary file 1 — Supporting Information S1 [file JFA2-18-e70089-s002.docx]

**Table 1.** SPIDER search tool and relevant search strategy.

| **Sample (S)** | **Phenomenon of interest (PI)** | **Design (D)** | **Evaluation (E)** | **Research type (R)** |
| --- | --- | --- | --- | --- |
| Human adults | Footwear needs  Footwear choice  Footwear design | Not specified | Understanding | Qualitative  Quantitative  Mixed methods |
| Limiters:  - Studies in human  - Age 18 years old or above | Search terms: “footwear need*” OR “footwear choice*” OR “footwear design*” | Not applicable | Not used as search term or limiters but used as inclusion criteria instead | No specific search strategy used as all research types are included |

**Table 2.** A summary of databases and search terms.

| **Date** | **Database** | **Search terms** |
| --- | --- | --- |
| 1/3/2022 | PubMed | (("footwear need*" AND ((humans[Filter]) AND (english[Filter]))) OR ("footwear choice*" AND ((humans[Filter]) AND (english[Filter])))) OR ("footwear design*" AND ((humans[Filter]) AND (english[Filter]))) |
| 1/3/2022 | Scopus | "footwear need*" OR "footwear choice*" OR "footwear design*" AND (LIMIT-TO (LANGUAGE, "English")) AND (LIMIT-TO (DOCTYPE, "ar") OR LIMIT-TO (DOCTYPE, "re")) AND (LIMIT-TO (SRCTYPE , "j" )) |
| 1/3/2022 | Web of Science (WoS) | TS=("footwear need*" OR "footwear choice*" OR "footwear design*")  Limiters - journal articles, journal reviews, English |
