# Supplementary material for: Understanding Footwear Needs: A Conceptual Review
Source: J Foot Ankle Res. 2025 Oct 11;18(4):e70089. doi: 10.1002/jfa2.70089 (PMC12515056; doi:10.1002/jfa2.70089)
Supplement: Supplementary file 2 — Supporting Information S2 [file JFA2-18-e70089-s001.docx]

**An overview of conceptual anatomy sections, themes, subthemes, and supporting literature**

| **Conceptual anatomy sections** | **Themes** | **Subthemes** | **Number of supporting literature** | **References** |
| --- | --- | --- | --- | --- |
| **Preconditions** | Person | Who they are at present (Self-identity) | 34 | (Anderson et al., 2017; Barnish et al., 2017; Branthwaite and Chockalingam, 2019; Brenton-Rule et al., 2019, 2014; Carter et al., 2016; Catalani, 2015; Chari et al., 2015; Curwen and Park, 2014; Davis et al., 2013; Donovan-Hall et al., 2019; Ferreira and Scaraboto, 2016; Goodacre and Candy, 2011; Hendry et al., 2015; Hockey et al., 2014; Janson et al., 2021; Jellema et al., 2019; Johnson et al., 2006; McRitchie et al., 2018; Mills et al., 2010; Nicholls et al., 2020, 2018; Ozdinc et al., 2019; Paton et al., 2014; Robinson, 2014; Silvester et al., 2010; Sterzing et al., 2014; Tan et al., 2019; Williams et al., 2010, 2007; Williams and Nester, 2006; Yick et al., 2019) |
|  |  | What are their past experiences | 24 | (Anderson et al., 2017; Apps et al., 2014; Arts et al., 2014; Bagheri et al., 2019; Barwick et al., 2018; Branthwaite and Chockalingam, 2019; Brenton-Rule et al., 2019; Chari et al., 2015; Donovan-Hall et al., 2019; Ferreira and Scaraboto, 2016; Hockey et al., 2014; Huang et al., 2018; McRitchie et al., 2018; Melvin et al., 2019; Norlander et al., 2015; Ozdinc et al., 2019; Paton et al., 2014, 2013; Punjabi and Bagchi, 2020; Puszczalowska-Lizis et al., 2021; Tan et al., 2019; Tehan et al., 2019; Williams et al., 2010; Williams and Nester, 2006) |
|  |  | Who they want to become (Future intentions) | 12 | (Catalani, 2015; Chari et al., 2015; Davis et al., 2013; Hockey et al., 2014; Reinschmidt and Nigg, 2000; Robinson, 2014; Sterzing et al., 2014; Tan et al., 2019; Tavares et al., 2020; Tian et al., 2021; Williams et al., 2010; Williams and Nester, 2006) |
|  | Product (Footwear) | Perceptions | 24 | (Alcántara et al., 2005a, 2005b; Anderson et al., 2017; Apps et al., 2014; Arezes et al., 2013; Arts et al., 2014; Brenton-Rule et al., 2019, p. 20, 2014; Curwen and Park, 2014; Donovan-Hall et al., 2019; Hockey et al., 2014; Menz and Bonanno, 2021; Nicholls et al., 2020; Norlander et al., 2015; Paton et al., 2014, 2013; Punjabi and Bagchi, 2020; Puszczalowska-Lizis et al., 2021; Tan et al., 2019; Tehan et al., 2019; Tian et al., 2021; Verma and Sharma, 2018; West et al., 2019; Williams and Nester, 2006) |
|  | Circumstances | Activity | 6 | (Alcántara et al., 2005b; Anderson et al., 2017; Chander et al., 2019; Copper et al., 2021; Frecklington et al., 2019; Norlander et al., 2015) |
|  |  | Environment | 5 | (Brenton-Rule et al., 2014; Carter et al., 2016; Hockey et al., 2014; Silvester et al., 2010; Tan et al., 2019) |
| **Attributes** | Physiological needs | Fit for foot | 54 | (Ahmed et al., 2020; Anderson et al., 2017, 2021; Arts et al., 2014; Bagheri et al., 2019; Barwick et al., 2018; Bergin et al., 2013; Blazer et al., 2018; Branthwaite and Chockalingam, 2019; Brenton-Rule et al., 2014, 2019; Carter et al., 2016; Collings et al., 2021; Copper et al., 2021; Curwen and Park, 2014; Donovan-Hall et al., 2019; Ellis et al., 2002; Ferreira and Scaraboto, 2016; Frecklington et al., 2019; Gao et al., 2008; Goodacre and Candy, 2011; Hatton and Rome, 2019; Hendry et al., 2015; Hockey et al., 2014; Huang et al., 2018; Igiri et al., 2018; Janson et al., 2021; Jellema et al., 2019; Johnson et al., 2006; Jones et al., 2019; Keukenkamp et al., 2021; López-Moral et al., 2019; Matthias et al., 2021; McPoil, 2000; McRitchie et al., 2018; Melvin et al., 2019; Mills et al., 2010; Nicholls et al., 2018, 2020; Paton et al., 2013; Punjabi and Bagchi, 2020; Puszczalowska-Lizis et al., 2021; Rahman, 2018; Reiber et al., 2002; Reinschmidt and Nigg, 2000; Riskowski et al., 2011; Rome et al., 2011; Silvester et al., 2010; Tehan et al., 2019; Tian et al., 2021; van der Zwaard et al., 2014; Vass et al., 2015; Williams et al., 2007) |
|  |  | Fit for purpose | 50 | (Ahmed et al., 2020; Anderson et al., 2021, 2017; Apps et al., 2014; Bagheri et al., 2019; Barwick et al., 2018; Blazer et al., 2018; Brenton-Rule et al., 2019, 2014; Chander et al., 2019; Chari et al., 2015; Copper et al., 2021; Donovan-Hall et al., 2019; Ellis et al., 2002; Frecklington et al., 2019; Gao et al., 2008; Goodacre and Candy, 2011; Hatton and Rome, 2019; Hendry et al., 2015; Hockey et al., 2014; Honert et al., 2020; Igiri et al., 2018; Janson et al., 2021; Keukenkamp et al., 2021; McPoil, 2000; McRitchie et al., 2018; Melvin et al., 2019; Menz and Bonanno, 2021; Nicholls et al., 2020, 2018; Norlander et al., 2015; Paton et al., 2014, 2013; Punjabi and Bagchi, 2020; Puszczalowska-Lizis et al., 2021; Ramsey et al., 2019; Reiber et al., 2002; Reinschmidt and Nigg, 2000; Riskowski et al., 2011; Rome et al., 2011; Shimazaki et al., 2016; Sterzing et al., 2014; Tan et al., 2019; Tehan et al., 2019; Tian et al., 2021; Vass et al., 2015; West et al., 2019; Williams et al., 2007; Williams and Nester, 2006; Yick et al., 2019) |
|  | Safety needs | Financial safety | 44 | (Alcántara et al., 2005a, 2005b; Anderson et al., 2017, 2021; Apps et al., 2014; Arezes et al., 2013; Arts et al., 2014; Bagheri et al., 2019; Bergin et al., 2013; Brenton-Rule et al., 2014; Carter et al., 2016; Catalani, 2015; Collings et al., 2021; Curwen and Park, 2014; Donovan-Hall et al., 2019; Frecklington et al., 2019; Gao et al., 2008; Hendry et al., 2015; Hockey et al., 2014; Huang et al., 2018; Janson et al., 2021; Jellema et al., 2019; Keukenkamp et al., 2021, 2022; McPoil, 2000; Menz and Bonanno, 2021; Nicholls et al., 2020; Norlander et al., 2015; Ozdinc et al., 2019; Punjabi and Bagchi, 2020; Puszczalowska-Lizis et al., 2021; Rahman, 2018; Reiber et al., 2002; Reinschmidt and Nigg, 2000; Robinson, 2014; Sterzing et al., 2014; van der Zwaard et al., 2014; Vass et al., 2015; Verma and Sharma, 2018) |
|  |  | Footwear safety | 34 | (Anderson et al., 2021, 2017; Apps et al., 2014; Bagheri et al., 2019; Brenton-Rule et al., 2019, 2014; Chander et al., 2019; Davis et al., 2013; Donovan-Hall et al., 2019; Ellis et al., 2002; Gao et al., 2008; Goodacre and Candy, 2011; Hatton and Rome, 2019; Igiri et al., 2018; Janson et al., 2021; Jellema et al., 2019; Keukenkamp et al., 2021; McPoil, 2000; McRitchie et al., 2018; Melvin et al., 2019; Menz and Bonanno, 2021; Norlander et al., 2015; Ozdinc et al., 2019; Paton et al., 2013; Punjabi and Bagchi, 2020; Puszczalowska-Lizis et al., 2021; Reinschmidt and Nigg, 2000; Rome et al., 2011; Tehan et al., 2019; Tian et al., 2021; van der Zwaard et al., 2014; Vass et al., 2015; Williams and Nester, 2006; Yick et al., 2019) |
|  | Social needs | Fit for person | 8 | (Curwen and Park, 2014; Davis et al., 2013; Donovan-Hall et al., 2019; McRitchie et al., 2018; Nicholls et al., 2020, 2018; Tehan et al., 2019; Williams et al., 2010) |
|  |  | Fit for society | 12 | (Barnish et al., 2017; Curwen and Park, 2014; Hockey et al., 2014; Johnson et al., 2006; McRitchie et al., 2018; Menz and Bonanno, 2021; Nicholls et al., 2020; Robinson, 2014; Tan et al., 2019; Tehan et al., 2019; Williams et al., 2010, 2007) |
|  | Emotional needs | - | 4 | (Catalani, 2015; Curwen and Park, 2014; McRitchie et al., 2018; Tehan et al., 2019) |

|  | Adjunct attributes | Competing demands | 24 | (Bagheri et al., 2019; Copper et al., 2021; Curwen and Park, 2014; Donovan-Hall et al., 2019; Frecklington et al., 2019; Goodacre and Candy, 2011; Hatton and Rome, 2019; Hockey et al., 2014; Johnson et al., 2006; Nicholls et al., 2020, 2018; Norlander et al., 2015; Paton et al., 2014; Puszczalowska-Lizis et al., 2021; Reinschmidt and Nigg, 2000; Riskowski et al., 2011; Robinson, 2014; Sayadi et al., 2019; Silvester et al., 2010; Sterzing et al., 2014; Tan et al., 2019; Tehan et al., 2019; Williams et al., 2010, 2007) |
| --- | --- | --- | --- | --- |
| **Outcomes** | Physical | - | 59 | (Ahmed et al., 2020; Anderson et al., 2017; Arts et al., 2014; Bagheri et al., 2019; Barnish et al., 2017; Barwick et al., 2018; Bergin et al., 2013; Blazer et al., 2018; Branthwaite and Chockalingam, 2019; Brenton-Rule et al., 2019, 2014; Catalani, 2015; Chander et al., 2019; Chari et al., 2015; Collings et al., 2021; Copper et al., 2021; Davis et al., 2013; Donovan-Hall et al., 2019; Ellis et al., 2002; Frecklington et al., 2019; Gimunová et al., 2017; Goodacre and Candy, 2011; Hatton and Rome, 2019; Hendry et al., 2015; Honert et al., 2020; Huang et al., 2018; Igiri et al., 2018; Janson et al., 2021; Jellema et al., 2019; Jones et al., 2019; Keukenkamp et al., 2022, 2021; McPoil, 2000; McRitchie et al., 2018; Melvin et al., 2019; Menz and Bonanno, 2021; Nicholls et al., 2020, 2018; Norlander et al., 2015; Ozdinc et al., 2019; Paton et al., 2013; Puszczalowska-Lizis et al., 2021; Rahman, 2018; Ramsey et al., 2019; Riskowski et al., 2011; Rome et al., 2011; Shimazaki et al., 2016; Silvester et al., 2010; Tan et al., 2019; Tavares et al., 2020; Tehan et al., 2019; Tian et al., 2021; van der Zwaard et al., 2014; Vass et al., 2015; Williams et al., 2010, 2007; Williams and Nester, 2006; Yick et al., 2019) |
|  | Psychological | - | 27 | (Alcántara et al., 2005a; Arts et al., 2014; Barnish et al., 2017; Brenton-Rule et al., 2019, 2014; Carter et al., 2016; Curwen and Park, 2014; Davis et al., 2013; Donovan-Hall et al., 2019; Ferreira and Scaraboto, 2016; Frecklington et al., 2019, 2019; Goodacre and Candy, 2011; Hatton and Rome, 2019; Hendry et al., 2015; Janson et al., 2021; Johnson et al., 2006; Keukenkamp et al., 2022, 2021; Nicholls et al., 2018; Rahman, 2018; Tan et al., 2019; Tehan et al., 2019; Verma and Sharma, 2018; Williams et al., 2010, 2007; Williams and Nester, 2006; Yick et al., 2019) |
|  | Social | - | 4 | (Brenton-Rule et al., 2019; Donovan-Hall et al., 2019; Jellema et al., 2019; Puszczalowska-Lizis et al., 2021) |

**NB**: Each article can contain statements relevant to multiple sections of the anatomy of concept, in which case the article is referenced in all relevant sections.

**References**

Ahmed, S., Barwick, A., Butterworth, P., Nancarrow, S., 2020. Footwear and insole design features that reduce neuropathic plantar forefoot ulcer risk in people with diabetes: A systematic literature review. Journal of Foot and Ankle Research 13, 1–13. https://doi.org/10.1186/S13047-020-00400-4/TABLES/4

Alcántara, E., Artacho, M.A., González, J.C., García, A.C., 2005a. Application of product semantics to footwear design. Part I - Identification of footwear semantic space applying diferential semantics. International Journal of Industrial Ergonomics 35, 713–725. https://doi.org/10.1016/J.ERGON.2005.02.005

Alcántara, E., Artacho, M.A., González, J.C., García, A.C., 2005b. Application of product semantics to footwear design. Part II - Comparison of two clog designs using individual and compared semantic profiles. International Journal of Industrial Ergonomics 35, 727–735. https://doi.org/10.1016/J.ERGON.2005.02.006

Anderson, J., Williams, A.E., Nester, C., 2021. Musculoskeletal disorders, foot health and footwear choice in occupations involving prolonged standing. International Journal of Industrial Ergonomics 81, 103079. https://doi.org/10.1016/j.ergon.2020.103079

Anderson, J., Williams, A.E., Nester, C., 2017. An explorative qualitative study to determine the footwear needs of workers in standing environments. Journal of Foot and Ankle Research 10, 1–10. https://doi.org/10.1186/s13047-017-0223-4

Apps, C., Liu, H., Pykett, J., Sterzing, T., 2014. Gym training shoe requirements in China and England. Footwear Science 7, 51–62. https://doi.org/10.1080/19424280.2014.983446

Arezes, P.M., Neves, M.M., Teixeira, S.F., Leao, C.P., Cunha, J.L., 2013. Testing thermal comfort of trekking boots: an objective and subjective evaluation. Applied Ergonomics 44, 557–565. https://doi.org/10.1016/j.apergo.2012.11.007

Arts, M.L.J., De Haart, M., Bus, S.A., Bakker, J.P.J., Hacking, H.G.A., Nollet, F., 2014. Perceived usability and use of custom-made footwear in diabetic patients at high risk for foot ulceration. Journal of Rehabilitation Medicine 46, 357–362. https://doi.org/10.2340/16501977-1272

Bagheri, Z.S., Patel, N., Li, Y., Morrone, K., Fernie, G., Dutta, T., 2019. Slip resistance and wearability of safety footwear used on icy surfaces for outdoor municipal workers. Work 62, 37–47. https://doi.org/10.3233/WOR-182840

Barnish, M., Morgan, H.M., Barnish, J., 2017. The 2016 HIGh Heels: Health effects and psychosexual BenefITS (HIGH HABITS) study: Systematic review of reviews and additional primary studies. BMC Public Health 18, 1–13. https://doi.org/10.1186/S12889-017-4573-4

Barwick, A.L., van Netten, J.J., Reed, L.F., Lazzarini, P.A., 2018. Independent factors associated with wearing different types of outdoor footwear in a representative inpatient population: A cross-sectional study. Journal of Foot and Ankle Research 11, 1–8. https://doi.org/10.1186/S13047-018-0260-7

Bergin, S.M., Nube, V.L., Alford, J.B., Allard, B.P., Gurr, J.M., Holland, E.L., Horsley, M.W., Kamp, M.C., Lazzarini, P.A., Sinha, A.K., Warnock, J.T., Wraight, P.R., 2013. Australian Diabetes Foot Network: practical guideline on the provision of footwear for people with diabetes. Journal of Foot and Ankle Research 6, 6. https://doi.org/10.1186/1757-1146-6-6

Blazer, M.M., Jamrog, L.B., Schnack, L.L., 2018. Does the Shoe Fit? Considerations for Proper Shoe Fitting. Orthopaedic Nursing 37, 169–174. https://doi.org/10.1097/NOR.0000000000000447

Branthwaite, H., Chockalingam, N., 2019. Everyday footwear: An overview of what we know and what we should know on ill-fitting footwear and associated pain and pathology. Foot (Edinburgh, Scotland) 39, 11–14. https://doi.org/10.1016/j.foot.2019.01.007

Brenton-Rule, A., Dalbeth, N., Edwards, N.L., Rome, K., 2019. Experience of finding footwear and factors contributing to footwear choice in people with gout: A mixed methods study using a web-based survey. Journal of Foot and Ankle Research 12, 1–8. https://doi.org/10.1186/S13047-018-0313-Y

Brenton-Rule, A., Hendry, G.J., Barr, G., Rome, K., 2014. An evaluation of seasonal variations in footwear worn by adults with inflammatory arthritis: A cross-sectional observational study using a web-based survey. Journal of Foot and Ankle Research 7, 1–7. https://doi.org/10.1186/S13047-014-0036-7

Carter, K., Lahiri, M., Cheung, P.P., Santosa, A., Rome, K., 2016. Footwear characteristics in people with inflammatory arthritis in Singapore. Journal of Foot and Ankle Research 9, 1–5. https://doi.org/10.1186/S13047-016-0161-6

Catalani, A., 2015. Fashionable Curiosities: Extreme Footwear as Wearable Fantasies. Fashion Theory 19, 565–582. https://doi.org/10.1080/1362704X.2015.1071068

Chander, H., Knight, A.C., Carruth, D., 2019. Does Minimalist Footwear Design Aid in Postural Stability and Fall Prevention in Ergonomics?: Ergonomics in Design: The Quaterly of Human Factors Applications 27, 22–25. https://doi.org/10.1177/1064804619843384

Chari, S.R., McRae, P., Stewart, M.J., Webster, J., Fenn, M., Haines, T.P., Chari, S.R., McRae, P., Stewart, M.J., Webster, J., Fenn, M., Haines, T.P., 2015. Point prevalence of suboptimal footwear features among ambulant older hospital patients: implications for fall prevention. Australian Health Review 40, 399–404. https://doi.org/10.1071/AH14168

Collings, R., Freeman, J., Latour, J.M., Paton, J., 2021. Footwear and insole design features for offloading the diabetic at risk foot—A systematic review and meta-analyses. Endocrinology, Diabetes & Metabolism 4, e00132. https://doi.org/10.1002/EDM2.132

Copper, A.W., Scharfbillig, R., Nguyen, T.P., Collins, C., 2021. Identifying lower limb problems and the types of safety footwear worn in the Australian wine industry: a cross-sectional survey. Journal of Foot and Ankle Research 14, 1–10. https://doi.org/10.1186/s13047-021-00495-3

Curwen, L.G., Park, J., 2014. When the shoe doesn’t fit: Female consumers’ negative emotions. Journal of Fashion Marketing and Management 18, 338–356. https://doi.org/10.1108/JFMM-12-2012-0078

Davis, A., Murphy, A., Haines, T.P., 2013. “Good for older ladies, not me”: how elderly women choose their shoes. Journal of the American Podiatric Medical Association 103, 465–470. https://doi.org/10.7547/1030465

Donovan-Hall, M., Robison, J., Cole, M., Ashburn, A., Bowen, C., Burnett, M., Mamode, L., Pickering, R., Bader, D., Kunkel, D., 2019. The trouble with footwear following stroke: a qualitative study of the views and experience of people with stroke. Disability and Rehabilitation 42, 1107–1114. https://doi.org/10.1080/09638288.2018.1516816

Ellis, M.R., Campbell, J.D., Detwiler-Breidenbach, A., Hubbard, D.K., 2002. What do family physicians think about spirituality in clinical practice? The Journal Of Family Practice 51, 249–254.

Ferreira, M.C., Scaraboto, D., 2016. “My plastic dreams”: Towards an extended understanding of materiality and the shaping of consumer identities. Journal of Business Research 69, 191–207. https://doi.org/10.1016/J.JBUSRES.2015.07.032

Frecklington, M., Williams, A., Dalbeth, N., McNair, P., Gow, P., Rome, K., 2019. The footwear experiences of people with gout: A qualitative study. Journal of Foot and Ankle Research 12, 1–6. https://doi.org/10.1186/S13047-019-0349-7

Gao, C., Holmér, I., Abeysekera, J., 2008. Slips and falls in a cold climate: Underfoot surface, footwear design and worker preferences for preventive measures. Applied Ergonomics 39, 385–391. https://doi.org/10.1016/J.APERGO.2007.08.001

Gimunová, M., Zvonař, M., Kolářová, K., Janík, Z., Mikeska, O., Musil, R., Ventruba, P., Šagat, P., 2017. Changes in lower extremity blood flow during advancing phases of pregnancy and the effects of special footwear. Jornal Vascular Brasileiro 16, 214–219. https://doi.org/10.1590/1677-5449.002617

Goodacre, L.J., Candy, F.J., 2011. ‘If I didn’t have RA I wouldn’t give them house room’: the relationship between RA, footwear and clothing choices. Rheumatology 50, 513–517. https://doi.org/10.1093/RHEUMATOLOGY/KEQ347

Hatton, A.L., Rome, K., 2019. Falls, Footwear, and Podiatric Interventions in Older Adults. Clinics in Geriatric Medicine 35, 161–171. https://doi.org/10.1016/J.CGER.2018.12.001

Hendry, G.J., Brenton-Rule, A., Barr, G., Rome, K., 2015. Footwear Experiences of People With Chronic Musculoskeletal Diseases. Arthritis Care & Research 67, 1164–1172. https://doi.org/10.1002/ACR.22548

Hockey, J., Dilley, R., Robinson, V., Sherlock, A., 2014. The Temporal Landscape of Shoes: A Life Course Perspective: The Sociological Review 62, 255–275. https://doi.org/10.1111/1467-954X.12154

Honert, E.C., Mohr, M., Lam, W.K., Nigg, S., 2020. Shoe feature recommendations for different running levels: A Delphi study. PLOS ONE 15, e0236047. https://doi.org/10.1371/JOURNAL.PONE.0236047

Huang, S., Wang, Z., Jiang, Y., 2018. Guess your size: A hybrid model for footwear size recommendation. Advanced Engineering Informatics 36, 64–75. https://doi.org/10.1016/j.aei.2018.02.003

Igiri, B.E., Tagang, J.I., Okoduwa, S.I.R., Adeyi, A.O., Okeh, A., 2018. An integrative review of therapeutic footwear for neuropathic foot due to diabetes mellitus. Diabetes & Metabolic Syndrome: Clinical Research & Reviews 13. https://doi.org/10.1016/j.dsx.2018.12.011

Janson, D., Newman, S.T., Dhokia, V., 2021. Safety footwear: A survey of end-users. Applied Ergonomics 92, 103333. https://doi.org/10.1016/J.APERGO.2020.103333

Jellema, A.H., Huysmans, T., Hartholt, K., van der Cammen, T.J.M., 2019. Shoe design for older adults: Evidence from a systematic review on the elements of optimal footwear. Maturitas 127, 64–81. https://doi.org/10.1016/J.MATURITAS.2019.06.002

Johnson, M., Newton, P., Goyder, E., 2006. Patient and professional perspectives on prescribed therapeutic footwear for people with diabetes: A vignette study. Patient Education and Counseling 64, 167–172. https://doi.org/10.1016/J.PEC.2005.12.013

Jones, P.J., Bibb, R.J., Davies, M.J., Khunti, K., McCarthy, M., Fong, D.T.P., Webb, D., 2019. A fitting problem: Standardising shoe fit standards to reduce related diabetic foot ulcers. Diabetes Research and Clinical Practice 154, 66–74. https://doi.org/10.1016/J.DIABRES.2019.05.017

Keukenkamp, R., Van Netten, J.J., Busch-Westbroek, T.E., Bus, S.A., 2022. Custom-made footwear designed for indoor use increases short-term and long-term adherence in people with diabetes at high ulcer risk. BMJ Open Diabetes Research and Care 10, e002593. https://doi.org/10.1136/BMJDRC-2021-002593

Keukenkamp, R., van Netten, J.J., Busch-Westbroek, T.E., Nollet, F., Bus, S.A., 2021. Users’ needs and expectations and the design of a new custom-made indoor footwear solution for people with diabetes at risk of foot ulceration. Disability and Rehabilitation. https://doi.org/10.1080/09638288.2021.2003878

López-Moral, M., Lázaro-Martínez, J.L., García-Morales, E., García-Álvarez, Y., JavierÁlvaro-Afonso, F., Molines-Barroso, R.J., 2019. Clinical efficacy of therapeutic footwear with a rigid rocker sole in the prevention of recurrence in patients with diabetes mellitus and diabetic polineuropathy: A randomized clinical trial. PLOS ONE 14, e0219537. https://doi.org/10.1371/JOURNAL.PONE.0219537

Matthias, E.C., Banwell, H.A., Arnold, J.B., 2021. Methods for assessing footwear comfort: a systematic review. Footwear Science 13, 255–274. https://doi.org/10.1080/19424280.2021.1961879

McPoil, T.G., 2000. Athletic footwear: Design, performance and selection issues. Journal of Science and Medicine in Sport 3, 260–267. https://doi.org/10.1016/S1440-2440(00)80035-3

McRitchie, M., Branthwaite, H., Chockalingam, N., 2018. Footwear choices for painful feet - an observational study exploring footwear and foot problems in women. Journal of Foot and Ankle Research 11, 1–7. https://doi.org/10.1186/s13047-018-0265-2

Melvin, J.M.A., Price, C., Preece, S., Nester, C., Howard, D., 2019. An investigation into the effects of, and interaction between, heel height and shoe upper stiffness on plantar pressure and comfort. Footwear Science 11, 25–34. https://doi.org/10.1080/19424280.2018.1555862

Menz, H.B., Bonanno, D.R., 2021. Footwear comfort: a systematic search and narrative synthesis of the literature. Journal of Foot and Ankle Research 2021 14:1 14, 1–11. https://doi.org/10.1186/S13047-021-00500-9

Mills, K., Blanch, P., Vicenzino, B., 2010. Identifying clinically meaningful tools for measuring comfort perception of footwear. Medicine and Science in Sports and Exercise 42, 1966–1971. https://doi.org/10.1249/MSS.0b013e3181dbacc8

Nicholls, E., Robinson, V., Farndon, L., Vernon, W., 2020. “You don’t like to tell them their job but it’s your foot at the end of the day”: theorising and negotiating ‘resistance’ in clinical encounters. Social Theory & Health 2020 19:3 19, 246–262. https://doi.org/10.1057/S41285-020-00134-0

Nicholls, E., Robinson, V., Farndon, L., Vernon, W., 2018. “A good fit?” Bringing the sociology of footwear to the clinical encounter in podiatry services: A narrative review. Journal of Foot and Ankle Research 11, 1–5. https://doi.org/10.1186/S13047-018-0253-6

Norlander, A., Miller, M., Gard, G., 2015. Perceived risks for slipping and falling at work during wintertime and criteria for a slip-resistant winter shoe among Swedish outdoor workers. Safety Science 73, 52–61. https://doi.org/10.1016/J.SSCI.2014.11.009

Ozdinc, S., Unsar, S., Kostak, M.A., 2019. Musculoskeletal problems and attitudes towards footwear among university students. Journal of Back and Musculoskeletal Rehabilitation 32, 141–147. https://doi.org/10.3233/BMR-171036

Paton, J., Roberts, A., Bruce, G.K., Marsden, J., 2014. Patients’ Experience of therapeutic footwear whilst living at risk of neuropathic diabetic foot ulceration: An interpretative phenomenological analysis (IPA). Journal of Foot and Ankle Research 7, 1–9. https://doi.org/10.1186/1757-1146-7-16

Paton, J.S., Roberts, A., Bruce, G.K., Marsden, J., 2013. Does Footwear Affect Balance? Journal of the American Podiatric Medical Association. https://doi.org/10.7547/1030508

Punjabi, M., Bagchi, A., 2020. Brand loyalty and sports attributes preference in basketball Brand Loyalty and Shoe Attributes Preference of Indian National Basketball Players. Annals of Tropical Medicine & Public Health http 23. https://doi.org/10.36295/ASRO.2020.231728

Puszczalowska-Lizis, E., Koziol, K., Omorczyk, J., 2021. Perception of footwear comfort and its relationship with the foot structure among youngest-old women and men. PeerJ 9, e12385. https://doi.org/10.7717/PEERJ.12385

Rahman, O., 2018. Online Consumer Choice: Footwear Design and Visual Presentation. Journal of Textile and Apparal, Technology and Management 10.

Ramsey, C.A., Lamb, P., Kaur, M., Baxter, G.D., Ribeiro, D.C., 2019. “How are running shoes assessed? A systematic review of characteristics and measurement tools used to describe running footwear.” Journal of Sports Sciences 37, 1617–1629. https://doi.org/10.1080/02640414.2019.1578449

Reiber, G.E., Smith, D.G., Wallace, C.M., Vath, C.A., Sullivan, K., Hayes, S., Yu, O., Martin, D., Maciejewski, M., 2002. Footwear used by individuals with diabetes and a history of foot ulcer. Journal of Rehabilitation Research and Development 39, 615–622.

Reinschmidt, C., Nigg, B.M., 2000. Current Issues in the Design of Running and Court Shoes. Sportverletzung · Sportschaden 14, 72–81. https://doi.org/10.1055/s-2000-7866

Riskowski, J., Dufour, A.B., Hannan, M.T., 2011. Arthritis, Foot Pain & Shoe Wear: Current Musculoskeletal Research on Feet. Current opinion in rheumatology 23, 148. https://doi.org/10.1097/BOR.0B013E3283422CF5

Robinson, V., 2014. Risky footwear practices: masculinity, identity and crisis. International Journal for Masculinity Studies 9, 151–165. https://doi.org/10.1080/18902138.2014.950501

Rome, K., Frecklington, M., McNair, P., Gow, P., Dalbeth, N., 2011. Footwear characteristics and factors influencing footwear choice in patients with gout. Arthritis Care & Research 63, 1599–1604. https://doi.org/10.1002/acr.20582

Sayadi, J., Sayadi, L.R., Fijany, A., Chopan, M., Hermanowicz, N., 2019. Assessing and addressing footwear needs in Parkinson’s disease-design thinking in neurology. NeuroRehabilitation 45, 443–448. https://doi.org/10.3233/NRE-192897

Shimazaki, Y., Matsutani, T., Satsumoto, Y., 2016. Evaluation of thermal formation and air ventilation inside footwear during gait: The role of gait and fitting. Applied Ergonomics 55, 234–240. https://doi.org/10.1016/J.APERGO.2015.11.002

Silvester, R.N., Williams, A.E., Dalbeth, N., Rome, K., 2010. “Choosing shoes”: A preliminary study into the challenges facing clinicians in assessing footwear for rheumatoid patients. Journal of Foot and Ankle Research 3, 1–8. https://doi.org/10.1186/1757-1146-3-24

Sterzing, T., Barnes, S., Althoff, K., Determan, L., Liu, H., Cheung, J.T.M., 2014. Tennis shoe requirements in China, USA, and Germany. Footwear Science 6, 165–176. https://doi.org/10.1080/19424280.2014.924590

Tan, S., Horobin, H., Tunprasert, T., 2019. The lived experience of people with diabetes using off-the-shelf prescription footwear in Singapore: A qualitative study using interpretative phenomenological analysis. Journal of Foot and Ankle Research 12, 1–12. https://doi.org/10.1186/S13047-019-0329-Y

Tavares, J., Jost, T., Drewelow, G., Rylander, J., 2020. Do maximalist shoes mitigate risk factors for tibial stress fractures better than stability or flexible (marketed as minimalist) shoes? Footwear Science 12, 63–74. https://doi.org/10.1080/19424280.2019.1708977

Tehan, P.E., Morpeth, T., Williams, A.E., Dalbeth, N., Rome, K., 2019. “Come and live with my feet and you’ll understand” - A qualitative study exploring the experiences of retail footwear in women with rheumatoid arthritis. Journal of Foot and Ankle Research 12, 1–9. https://doi.org/10.1186/S13047-019-0328-Z

Tian, M., Lei, Y., Li, J., 2021. A Triangle Design Framework for Functional Footwear for Chinese Older Adults. Fashion Practice 13, 69–87. https://doi.org/10.1080/17569370.2021.1872898

van der Zwaard, B.C., Poppe, E., Vanwanseele, B., van der Horst, H.E., Elders, P.J.M., 2014. Development and evaluation of a leaflet containing shoe advice: a randomized controlled trial. Family Practice 31, 267–272. https://doi.org/10.1093/FAMPRA/CMT084

Vass, C., Edwards, C., Smith, A., Sahota, O., Drummond, A., 2015. What do patients wear on their feet? A service evaluation of footwear in elderly patients. International Journal of Therapy and Rehabilitation 22, 225–232. https://doi.org/10.12968/IJTR.2015.22.5.225

Verma, P., Sharma, A.K., 2018. Assortment satisfaction: The tale of online footwear sales. Technology in Society 54, 57–65. https://doi.org/10.1016/J.TECHSOC.2018.03.004

West, A.M., Tarrier, J., Hodder, S., Havenith, G., 2019. Sweat distribution and perceived wetness across the human foot: the effect of shoes and exercise intensity. Ergonomics 62, 1450–1461. https://doi.org/10.1080/00140139.2019.1657185

Williams, A.E., Nester, C.J., 2006. Patient perceptions of stock footwear design features. Prosthetics and Orthotics International 30, 61–71. https://doi.org/10.1080/03093640600574425

Williams, A.E., Nester, C.J., Ravey, M.I., Kottink, A., Klapsing, M.G., 2010. Women’s experiences of wearing therapeutic footwear in three European countries. Journal of Foot and Ankle Research 3, 1–8. https://doi.org/10.1186/1757-1146-3-23

Williams, A.E., Rome, K., Nester, C.J., 2007. A clinical trial of specialist footwear for patients with rheumatoid arthritis. Rheumatology 46, 302–307. https://doi.org/10.1093/RHEUMATOLOGY/KEL234

Yick, K. lun, Yu, A., Li, P. ling, 2019. Insights into footwear preferences and insole design to improve thermal environment of footwear. International Journal of Fashion Design, Technology and Education 12, 325–334. https://doi.org/10.1080/17543266.2019.1629028
